# Effects of dietary *Enteromorpha* powder on reproduction-related hormones and genes during the late laying period of Zi geese

**DOI:** 10.5713/ajas.20.0368

**Published:** 2020-08-30

**Authors:** Wei Qing Ma, Dan Hua Zhao, Huang Zuo Cheng, Si Bo Wang, Ji Yang, Hong Xia Cui, Ming Yuan Lu, Hong Zhi Wu, Li Xu, Guo Jun Liu

**Affiliations:** 1 College of Animal Science and Technology, Northeast Agricultural University, Harbin 150030, China; 2 Institute of Animal Husbandry of Heilongjiang Academy of Agricultural Sciences, Harbin 150086, China

**Keywords:** *Enteromorpha* Powder, Hormones, Genes, Late Laying Period, Zi Geese

## Abstract

**Objective:**

The aim of this study was to investigate the effects of *Enteromorpha* powder supplementation on reproduction-related hormones and genes in the late laying period of Zi geese.

**Methods:**

A total of 312 (1-year-old) Zi geese with similar laying rate were randomly divided into 2 groups with 6 replicates each, each with 21 female geese and 5 male geese. The control group was fed with a basal diet and the test group was fed with a diet containing 3% *Enteromorpha* powder. The trial period lasted for 7 weeks.

**Results:**

Our results showed that the laying rate was improved in the test group at each week of trial (p<0.01), and the levels of estradiol in serum and prolactin in ovary were increased compared with the control group (p<0.05).

**Conclusion:**

Based on above results, *Enteromorpha* powder supplementation at 3% could promote reproductive performance during the late laying period of Zi geese.

## INTRODUCTION

*Enteromorpha* is a large green seaweed which reproduces seasonally and can explode in population during summers. Because it is rich in trace elements and biologically active substances, its study has gradually increased. Abudabos et al [1] found that replacing 3% corn with green seaweed in the diet not only improved the performance of broilers, but also reduced serum total lipid, cholesterol, and uric acid concentrations. *Enteromorpha* polysaccharide, which is a sort of sulfated polysaccharide extracted from *Enteromorpha* that has antioxidant, antibacterial, and anticancer effects, is used in the fields of cosmetics, food, and medicine [2]. However, the effects of *Enteromorpha* or its extracts on reproductive hormones had rarely been reported. It has generally been believed that serum levels of reproductive hormones reflect the body’s ability to reproduce. Egg laying is the result of multiple reproductive hormones acting on the ovary, which are in turn regulated by the hypothalamic-pituitary-gonadal (HPG) axis. Gonadotropin-releasing hormone (GnRH) released by the hypothalamus acts on the pituitary to produce gonadotropin. Follicle stimulating hormone (FSH) plays an important role in promoting follicular granulosa cell proliferation. Luteinizing hormone (LH) acts on ovaries to stimulate follicular maturation and ovulation. FSH and LH together regulate follicular synthesis of estradiol (E2) and progesterone (P4) [3–5]. With age, physiological changes reduce both the number and the quality of follicles, resulting in a decrease in egg production and reproductive performance in the late laying period [6–8]. Geese are poultry with low reproductive performance [9], therefore, it is important to study their reproductive hormones and genes in the late laying period. This experiment, which was inspired by Abudabos et al [1], substituted some basal feed ingredients with 3% *Enteromorpha* powder to improve the reproductive performance of Zi geese.

## MATERIALS AND METHODS

### Animal care

This experiment was conducted in accordance with the Chinese guidelines for animal welfare and with the animal welfare standards of the College of Animal Science and Technology, Northeast Agricultural University (NEAU-2018-0232).

### Experimental design

A total of 312 (1-year-old) Zi geese with similar laying rate were randomly allocated into two groups, the control group and the test group, each group had 6 replicates, 21 female geese and 5 male geese in each replicate. The control group was fed with basal diet and the test group was fed with basal diet containing 3% *Enteromorpha* powder (Table 1). *Enteromorpha* powder was purchased from Zhongtaihe Biotechnology Co., Ltd., Qingdao, China. *Enteromorpha* powder contains 6.64% crude protein, 6.00% crude fiber, 0.10% methionine (Met), 0.18% lysine (Lys). All geese were free to eat and drink, and additionally hydrated when the weather was hot. The feed was granular and the straw was used as bedding in the house. According to feeding management, the goose farm adopted natural light. The egg production was recorded daily, and the laying rate was calculated. The feeding trial was carried out at Zi geese breeding base of the Heilongjiang Academy of Agricultural Sciences. The feeding trial lasted 7 weeks after a 1-week adaptation period.

### Sample collection and analysis

Two female geese from each replication were randomly selected for blood sampling at the end of the fifth week of the experiment. Blood was collected after fasting for 12 h and centrifuged at 4,000 r/min for 10 min, and the supernatant was collected for analysis. At the end of the experiment, one female goose was selected from reah replication for slaughter, the ovary was taken and stored at −80°C, prior to testing. Both serum and ovary GnRH, FSH, LH, prolactin (PRL), E2, and P4 levels were determined by enzyme-linked immunosorbent assay using kits purchased from Shanghai Youxuan Biotechnology Co., Ltd., Shanghai, China.

### Quantification of *GnRH1*, *LHR*, and *ESR2* genes with real-time polymerase chain reaction

After the slaughter, the hypothalamus and ovary were carefully removed from female geese, and rapidly preservated at −80°C for determining the relative expression of gonadotropin-releasing hormone 1 (GnRH1) in the hypothalamus, and luteinizing hormone receptor (LHR), and estrogen receptor 2 (ESR2) in the ovary. Primers were designed by Beacon Designer 7 based on goose *GnRH1*, *LHR*, and *ESR2* gene sequences registered in NCBI, and using the glyceraldehyde-3-phosphate dehydrogenase gene as the internal reference gene, as shown in Table 2. Total RNA was extracted from the hypothalamus and ovary using RnaEx reagent. The total volume of the real-time polymerase chain reaction (PCR) reaction system was 10 μL, and the reaction system was as follows: SYBR Green Mix 4.4 μL, each of upstream and downstream primers 0.3 μL, cDNA 5 μL. The real-time PCR reaction procedure was as follows: 95°C for 10 min, 1 cycle, 95°C for 10 s, 60°C for 34 s, and 40 cycles. The Ct values of the target genes and the internal reference genes were measured, and the relative expression levels of the antioxidant genes were calculated by the 2^−ΔΔCt^ method.

### Statistical analysis

Data were analyzed using two-tailed Student’s T-tests in SPSS 22.0 (SPSS Inc., Chicago, IL, USA). Results were expressed as mean±standard deviation. Data significance was considered at p<0.05 and highly significant at p<0.01.

## RESULTS

### Production performance

As shown in Table 3, the control group decreased rapidly from the first week to the third week (the laying rate was less than 5%), and the test group was close to suspended production at the sixth week. During the whole trial period, the test group had a higher laying rate than the control group (p<0.01).

### Reproductive hormone analysis

We measured serum reproductive hormones at the fifth week of the trial. As shown in Table 4, the content of E2 in serum from the test group was higher than that of the control group (p<0.05), however, there was no significant difference between the two groups in any other indicators (p>0.05).

At the end of the trial, we isolated geese ovaries and mea sured relevant hormone levels, which results are shown in Table 5. The content of PRL in the ovary from the test group was higher than the control group (p<0.05), and the other indicators were higher than the control group but did not reach a significant level (p>0.05).

### GnRH1, LHR, and ESR2 mRNA expression

The results of *GnRH1*, *LHR*, and *ESR2* gene expression were shown in Table 6, and all data in the test group were lower than the control group but did not reach a significant level (p>0.05).

## DISCUSSION

At the late laying period, the body’s ability to regulate homeostasis of the ovary is reduced [7,8], and environmental changes such as light, temperature, and stress can easily increase ovarian reactive oxygen species levels, reduce DNA stability and mitochondrial function, and release cytochromes. Cytochromes c in concert with apoptotic factors, leads to apoptosis of follicular granule cells, imbalance of reproductive hormone secretion on the HPG axis, resulting in decreased egg production [10–12]. Our results showed that the egg production rate of both the test group and the control group were negatively correlated with the time since the start of laying, indicating that ovaries had shrank over time and were nearing the discontinuation period by the end of the trial period. Kulshreshtha et al [13] reported that supplementation of 2% or 4% red seaweed to 78-wk-old laying hens produced no significant difference in laying rate between all of the groups in the first to third weeks of the trial, but the laying rate of the test group was significantly improved compared with the control group in the fourth week. Our results were similar in that in our Zi geese supplemented with 3% *Enteromorpha* powder, the egg production rate of the test group was significantly higher than the control group at 1 to 7 weeks. This indicated that the *Enteromorpha* powder could prolong the laying period of Zi geese and increase egg production, which possibly due to effects of active substances such as brassica polysaccharides and unsaturated fatty acids on regulating reproductive hormone regulation at the HPG axis [14–16].

Generally speaking, GnRH released from the hypothala mus acts on the pituitary to promote the release of LH and FSH, which in turn acts on ovarian granulosa cells and binds to the corresponding receptors, promoting the growth and development of follicles [17,18]. E2 is a steroid hormone secreted by follicular cells and granulosa cells, which can synergize with FSH to promote follicular development and to induce the peak in LH before ovulation [19]. Bluhm et al [20] found that serum LH, PRL, E2, and P4 levels in wild duck laying period were significantly higher than those in the early laying period. The present study found that serum E2 concentration of the test group was significantly higher than the control group, which indicated that E2 was one of the key hormones in laying eggs at the late laying period. E2 can also exert its physiological effects through negative feedback regulation of the HPG axis. Sun et al [21] reported that E2 enhanced L-type calcium channel currents in GnRH neurons through estrogen receptor 2 (ER2) and membrane receptor G-protein coupled receptor 30 and increased excitability of GnRH neurons. Previous studies had shown that E2 has two effects on PRL: one is by directly acting on the pituitary to promote PRL release, and the other is by acting on the hypothalamus to promote the secretion of PRL releasing factor [22]. Zhu et al [23] found that *Morinda officinalis* polysaccharide could enhance the Kiss 1-GPR54 signaling pathway in the hypothalamus of male rats with varicocele and promoted the synthesis and secretion of GnRH. PRL is a polypeptide hormone secreted by eosinophils in the anterior pituitary and regulated by the upstream hormone GnRH. Many studies had shown that PRL can inhibit the secretion of LH and FSH, leading to follicular atresia, which is a key factor in inducing nesting behavior in poultry. However, PRL can also promote the development and production of poultry follicles and positively regulate egg production at the early laying period [24]. Yang et al [25] studied the periodic changes of reproductive hormones in Yangzhou geese, which showed that the laying rate was positively correlated with PRL concentration. In this study, ovarian PRL concentration in the test group was significantly increased than that of the control group, although the serum concentration did not reach a significant level, but the test group was also higher than the control group. It indicated that *Enteromorpha* powder could increase the concentration of PRL in the ovary and maintain Zi geese staying in the laying state. It had been found that the expression of PRL in the pituitary and plasma was inconsistent. Ishida et al. [26] reported that the concentration of PRL differed between the pituitary and plasma, and the plasma PRL level began to increase 7 days after hatching while the PRL pituitary concentration remained unchanged. Similarly, in this experiment, it was found that E2 and PRL have different levels in serum and ovary, probably because they played a role in different tissues. The increase of E2 and PRL levels in Zi geese in our test group may have been due to the polyunsaturated fatty acids in *Enteromorpha* powder promoting the synthesis and secretion of E2 and PRL on the HPG axis.

GnRH1 is mainly secreted by hypothalamic arcuate nu cleus GnRH neurons, which can stimulate the release of gonadotropins from the pituitary gland [27]. Previous studies have shown that *GnRH* gene is related to the reproductive cycle and is expressed in hypothalamus and ovary. Compared with nesting stage, the expression of *GnRH* gene was significantly higher in the laying stage [28,29]. Schirman-Hildesheim et al [30] reported that the expression of GnRH1 mRNA in the ovaries of estrus mice could change with time, which might be related to ovulation. Tran et al. [31] found that unsaturated fatty acids increased GnRH mRNA expression through GPR120 mediated downstream PKC/MAPK and PI3K signaling. LHR is a binding site that mediates the role of LH in the ovary. When the two proteins combine, it stimulates the synthesis and secretion of corpus callosum and promotes gonadal development [32]. ESR2 is an important member of the nuclear transcription factor receptor superfamily and plays an important role in follicular development. In mice with knocked out *ER2* gene, the numbers of ovarian follicles and corpus luteum decreased, the number of follicles with atresia and apoptosis increased, and follicular maturation and ovulation were inhibited [33]. Our results showed that GnRH1 in the hypothalamus and ovarian *LHR* and *ESR2* gene expression in the test group showed lower levels but did not reach a significant level compared with the control group. It may be that the addition of *Enteromorpha* powder in diet improves the levels of E2 and PRL in the body, which in turn negatively regulates the HPG axis and decreases the expression of *GnRH1*, *LHR*, and *ESR2* genes.

In conclusion, dietary supplementation with 3% *Enteromorpha* powder significantly increased the egg production, serum E2 concentration, ovarian PRL concentration, and reproductive performance of female Zi geese.

## Figures and Tables

**Table 1:** The compositions of the diet and the nutritional level

Items	Control group	Test group
Ingredient		
Corn (%)	39.60	39.60
Corn gluten feed (%)	21.00	18.40
Corn germ meal (%)	20.00	19.60
Corn oil (%)	0.50	0.50
Soybean meal (%)	10.00	10.00
*Enteromorpha* powder (%)	0.00	3.00
Limestone (%)	3.50	3.50
Dicalcium phosphate (%)	0.90	0.90
Salt (%)	0.35	0.35
DL-methionine (%)	0.15	0.15
Lysine (%)	0.01	0.01
Choline chloride (%)	0.08	0.08
Zeolite powder (%)	3.52	3.52
Premix1) (%)	0.39	0.39
Total	100.00	100.00
Nutrients		
ME (MJ/kg)	10.04	10.04
CP (%)	15.59	15.41
Met (%)	0.38	0.38
Lys (%)	0.64	0.63
Ca (%)	1.64	1.68
Total P (%)	0.58	0.56

ME, metabolizable energy; CP, crude protein.

1) Premix provided the following per kg of diet: vitamin A, 15,000 IU; vitamin D_3_, 5,300 IU; vitamin E, 100 mg; vitamin K, 4 mg; vitamin B_1_, 2 mg; vitamin B_2_, 10 mg; vitamin B_6_, 10 mg; vitamin B_12_, 0.1 mg; niacin, 100 mg; pantothenic acid, 50 mg; folic acid, 2 mg; biotin, 0.3 mg; Fe, 120 mg; Cu, 20 mg; Zn, 100 mg; Mn, 600 mg, I, 3 mg, Se, 0.5 mg.

**Table 2:** Fluorescent quantitative primer information

Genes	Primer sequences (5′-3′)	Product length (bp)
*GnRH1*	F: CACAACATCCACATTCTCCTGA	113
	R: GCGTGCCTGGTGTTCATT	
*LHR*	F: CTGGCTTCTTCACCGTCTT	173
	R: CACCGCAATCAGGATGGA	
*ESR2*	F: ACATTGACAGCAGAACACAGT	187
	R: CAGGAGCAGACCATAACACATT	
*GAPDH*	F:TAGTGAAGGCTGCTGCTGAT	102
	R:AGGTGGAGGAATGGCTGTC	

*GnRH1*, gonadotropin-releasing hormone 1; *LHR*, luteinizing hormone receptor; *ESR2*, estrogen receptor 2; *GAPDH*, glyceraldehyde-3-phosphate dehydrogenase.

**Table 3:** Effects of *Enteromorpha* powder on the laying rate during the late laying period of Zi geese

Item	Control group1)	Test group2)
1 wk	13.04^B^±1.31	19.50^A^±0.45
2 wk	6.35^B^±0.55	12.81^A^±0.69
3 wk	4.76^B^±0.25	12.02^A^±0.60
4 wk	3.06^B^±0.29	9.52^A^±0.82
5 wk	2.38^B^±0.38	7.94^A^±0.45
6 wk	0.93^B^±0.24	5.16^A^±0.18
7 wk	1.06^B^±0.49	3.84^A^±0.32
1–7 wk	4.51^B^±0.11	10.11^A^±0.32

1) Control group, fed with basal diet.

2) Test group, fed with basal diet contained 3% *Enteromorpha* powder.

A,B Means within each row with different letters differ significantly (p<0.01).

**Table 4:** Effects of *Enteromorpha* powder on reproductive hormone in serum during the late laying period of Zi geese

Item	Control group1)	Test group2)
GnRH (mIU/mL)	54.73±3.93	53.82±3.49
PRL (mIU/L)	508.43±23.29	524.03±26.69
LH (ng/mL)	190.28±4.52	191.84±1.49
FSH (mIU/mL)	10.17±0.77	9.90±0.31
E2 (pg/mL)	251.09^b^±4.60	266.19^a^±2.96
P4 (ng/mL)	5.75±0.60	5.15±0.29

Data are presented as mean±standard error of the mean, n = 6.

GnRH, gonadotropin-releasing hormone; PRL, prolactin; LH, luteinizing hormone; FSH, follicle stimulating hormone; E2, estradiol; P4, progesterone.

1) Control group, fed with basal diet.

2) Test group, fed with basal diet contained 3% *Enteromorpha* powder.

a,b Means within each row with different letters differ significantly (p<0.05).

**Table 5:** Effects of *Enteromorpha* powder on reproductive hormone in ovary during the late laying period of Zi geese

Item	Control group1)	Test group2)
GnRH (mIU/mL)	56.61±7.15	58.92±5.37
PRL (mIU/L)	421.07^b^±11.56	499.12^a^±17.18
LH (ng/mL)	165.18±5.48	182.59±6.18
FSH (mIU/mL)	9.67±1.02	9.95±0.46
E2 (pg/mL)	253.89±15.32	267.75±13.44
P4 (ng/mL)	4.82±0.63	5.23±0.43

Data are presented as mean±standard error of the mean, n = 6.

GnRH, gonadotropin-releasing hormone; PRL, prolactin; LH, luteinizing hormone; FSH, follicle stimulating hormone; E2, estradiol; P4, progesterone.

1) Control group, fed with basal diet.

2) Test group, fed with basal diet contained 3% *Enteromorpha* powder

a,b Means within each row with different letters differ significantly (p<0.05).

**Table 6:** Effects of *Enteromorpha* powder on reproductive hormone gene during the late laying period of Zi geese

Item	Control group1)	Test group2)
GnRH1	1.00±0.08	0.81±0.10
LHR	1.00±0.05	0.92±0.10
ESR2	1.00±0.05	0.89±0.07

Data are presented as mean±standard error of the mean, n = 6.

GnRH1, gonadotropin-releasing hormone 1; LHR, luteinizing hormone receptor; ESR2, estrogen receptor 2.

1) Control group, fed with basal diet.

2) Test group, fed with basal diet contained 3% *Enteromorpha* powder.
